# The Profession in England


**Published:** 1860-04

**Authors:** 


### ARTICLE XI.

### *The Profession in England.**

At the close of another year, we anxiously look for the results which should accrue to our profession, now supposed to be awakened to the necessity of reform. The eyes of our brethren in America have been directed towards us since the irruption in 1856, and they have been constrained to witness much that is highly discreditable and damaging to the cause in hand. If anything, therefore, can be asserted to prove a tendency to the better, I feel assured it will not be unacceptable. It is our habit to assume somewhat upon the genius and professional respectability of our English dentists, and propound the doctrine, that better than they do not exist, or at the least, they are equal to any others, let the locality be where it may—but although this comes out strictly true in many instances, upon the broad scale such assertions will not hold. The dentists of this country, as a community, want education. Certain things we can do, and do well, but when it comes to the embodiment of research and high scientific attainments for practical use, I apprehend that I am not speaking falsely or uncharitably when I say that the large majority in our ranks are deficient. This may be doubted by some, and denied by others, and I may be reminded that my remarks are both unnecessary and gratuitous, but how can it be otherwise where there is no standard recognized, nor proof demanded of a practitioner's actual ability. My opinions are founded upon observation, as well as the remarkable readiness with which the proposal of uniting the profession into one body for educational purposes was received. When

* This communication was intended for insertion in the January No. of the Journal, but it did not reach us till after that No. was printed. We take pleasure in publishing it now.—EDS.

there was reason to hope that better times in this direction were dawning upon us, the thought was cheering to a degree. Unhappily, the brightness of those first rays were soon sadly marred, and in the hurly burly of last year one and all were compelled to exclaim, "chaos is come again." Out of that confusion, however, it is my pleasing duty to inform you that I believe we are emerging. Party strife has long been our distinguishing characteristic, and that strife has not subsided nor diminished. Formerly such feelings appeared to overrule the whole of our time, leaving nothing for sober reflection and improvement. Now it is evident that each side has determined to play out its part without relaxing a fraction of its individuality, but the difference between former times and the present is, that the opposition has taken a different turn, and assumed a different tone. Once it was, who should crush out the other's influence. Now each party is eager to outstrip the other by the proof of its power in doing most good. The Odontological Society has its slow but steady method of action. The College is quicker and more showy in its movements. For months and months the Society has taken every means to press its convictions of the necessity of the Royal College of Surgeons examining dentists. The council of the College have not been permitted to lose sight of that idea.

In the mean time the College of Dentists has been performing a series of diplomatic gyrations, only at last to fall back upon its original policy, which may be summed up in two words, independence and education.

The advantage of one course of conduct over the other is manifest, for the Society has gained its object, while the College has reaped nothing except a decided loss of members, many of whom have not been content to remain neutral, but have joined the opposing Society.

So long as a foundation for better things is laid, we might be content to place the "feeling" on either side in a subordinate position, or overlook it altogether, were it not that unity of action would secure the former, and at the same time do away with the unsightly spectacle of a useful and honorable profession fiercely divided against itself. As an independent viewer of the rivalry going on, I cannot over estimate the important fact that we now have a dental hospital, two dental schools, and an examining board at the Royal College of Surgeons—composed of surgeons and dentists—who will test and reward the proficient candidate for the new degree. If antagonism can thus be turned to good account, what should we not be permitted to anticipate from united friendly action.

It is perfectly true that the hospital and both the schools were originated with a large amount of opposition as the incentive. Your excellent convention is a severe reproof to our disjointed condition, but I fear it will be a long time ere the dentists of the United Kingdom will meet thus for mutual and general good, the profession here being more decidedly "split" than ever. Once we were apart because we did not know each other. Now we are asunder because we do know and cannot appreciate each other. This same understanding has taken some time to work out, and some ugly circumstances have turned up in the process. Unfortunately, in England we labor under the distinction of "class." Those who have obtained a high position in their profession look for and demand a certain amount of respect, and as a rule that is best displayed by a deferential distance being rigidly maintained. They will not be dragged into contact with their inferiors, and he who makes the attempt is sure to meet with a repulse; nevertheless, "*Ogni medaglio ha il suo reverso.*"

The College of Dentists still maintain their intention of applying to government for a charter, but I anticipate there will be a difficulty in obtaining it, now a door has been opened specially for the purpose they desire, in another direction. The Metropolitan School is in full swing, and is likely to be well supported. They have received a very strong stimulant to exertion in Mr. Robinson's munificient gift of twenty guineas to be awarded as a prize every second year, for the best thesis on a given subject relating to dental science. This prize has been made over to the school in *perpetuity,* and is much spoken of.

The London School of Dental Surgery is also progressing very favorably, several pupils having entered. The session commences so as to harmonize with the summer sessions of the various medical schools in the metropolis. The hospital, I can inform you on good authority, is prospering, and although I cannot anticipate the report of that institution—which will not be issued until the middle of January next—I do not hesitate to say that nearly two thousand patients will have been treated by the close of this month, and it is possible a still larger number will be recorded.

Up to this date the curiculum of the Royal College of Surgeons has not been issued, so that aspirants for the licentiateship in dental surgery—as I apprehend it will be termed—do not yet know what and how much will be required of them. This, however, is certain, that all who possess a fair amount of practical knowledge will have no very great difficulty in possessing the diploma.

I trust the new year may inaugurate a still better feeling for advancement in our professional attainments, and that before long I shall have the happiness to inform you that we have forgiven and forgotten one another's feelings and that peace and good will have secured a permanent triumph over those lower passions, of the manifestation of which he is the better man who is most ashamed.

P. S. Neither of our journals for this month have as yet made their appearance.

London, *December 15th,* 1859.

